# An RCT of a decision aid to support informed choices about taking aspirin to prevent colorectal cancer and other chronic diseases: a study protocol for the SITA (Should I Take Aspirin?) trial

**DOI:** 10.1186/s13063-021-05365-8

**Published:** 2021-07-15

**Authors:** Shakira Milton, Jennifer McIntosh, Finlay Macrae, Patty Chondros, Lyndal Trevena, Mark Jenkins, Fiona M. Walter, Natalie Taylor, Lucy Boyd, Sibel Saya, Napin Karnchanachari, Kitty Novy, Carmody Forbes, Javiera Martinez Gutierrez, Kate Broun, Sara Whitburn, Sarah McGill, George Fishman, Julie Marker, Max Shub, Jon Emery

**Affiliations:** 1 Centre for Cancer Research, University of Melbourne, Melbourne, Australia; 2 Department of General Practice, University of Melbourne, Melbourne, Australia; 3 Department of Software Systems & Cybersecurity, Monash University, Melbourne, Australia; 4 Colorectal Medicine and Genetics, The Royal Melbourne Hospital, Melbourne, Australia; 5 Department of Medicine, The University of Melbourne, Melbourne, Australia; 6 Faculty of Medicine and Health, School of Public Health, The University of Sydney, Sydney, Australia; 7 Melbourne School of Population and Global Health, University of Melbourne, Melbourne, Australia; 8 The Primary Care Unit, University of Cambridge, Cambridge, UK; 9 Behavioral and Implementation Research and Evaluation, Cancer Council NSW, New South Wales, Australia; 10 Faculty of Medicine and Health, University of Sydney, Sydney, Australia; 11 Department of Family Medicine, School of Medicine, Pontificia Universidad Católica de Chile, Santiago, Chile; 12 Early Detection and Immunisation, Prevention Department, Cancer Council Victoria, Melbourne, Australia; 13 Belmore Road Medical Clinic, Melbourne, Australia; 14 Cancer Screening and Prevention, Cancer Institute NSW, St Leonards, Australia; 15 Primary Care Collaborative Cancer Clinical Trials Group (PC4), Community Advisory Group, University of Melbourne, Melbourne, Australia

**Keywords:** Preventive medicine, General practice, Primary care, Cancer prevention, Bowel cancer, Colorectal cancer, Aspirin, Guideline implementation, Chemoprevention, Decision Aid, Informed decision making

## Abstract

**Background:**

Australian guidelines recommend that all people aged 50–70 years old actively consider taking daily low-dose aspirin (100–300 mg per day) for 2.5 to 5 years to reduce their risk of colorectal cancer (CRC). Despite the change of national CRC prevention guidelines, there has been no active implementation of the guidelines into clinical practice.

We aim to test the efficacy of a health consultation and decision aid, using a novel expected frequency tree (EFT) to present the benefits and harms of low dose aspirin prior to a general practice consultation with patients aged 50–70 years, on informed decision-making and uptake of aspirin.

**Methods:**

Approximately five to seven general practices in Victoria, Australia, will be recruited to participate. Patients 50–70 years old, attending an appointment with their general practitioner (GP) for any reason, will be invited to participate in the trial. Two hundred fifty-eight eligible participants will be randomly allocated 1:1 to intervention or active control arms using a computer-generated allocation sequence stratified by general practice, sex, and mode of trial delivery (face-to-face or teletrial).

There are two co-primary outcomes: informed decision-making at 1-month post randomisation, measured by the Multi-dimensional Measure of Informed Choice (MMIC), and self-reported daily use of aspirin at 6 months. Secondary outcomes include decisional conflict at 1-month and other behavioural changes to reduce CRC risk at both time points.

**Discussion:**

This trial will test the efficacy of novel methods for implementing national guidelines to support informed decision-making about taking aspirin in 50–70-year-olds to reduce the risk of CRC and other chronic diseases.

**Trial registration:**

The Australian New Zealand Clinical Trials Registry (ANZCTR) ACTRN12620001003965. Registered on 10 October 2020.

**Supplementary Information:**

The online version contains supplementary material available at 10.1186/s13063-021-05365-8.

## Administrative information

Note: the numbers in curly brackets in this protocol refer to SPIRIT checklist item numbers. The order of the items has been modified to group similar items (see http://www.equator-network.org/reporting-guidelines/spirit-2013-statement-defining-standard-protocol-items-for-clinical-trials/).

**Table:** 

Title {1}	An RCT of a decision aid to support informed choices about taking aspirin to prevent colorectal cancer and other chronic diseases: a study protocol for the SITA (Should I Take Aspirin?) trial
Trial registration {2a and 2b}.	ACTRN12620001003965 The Australian New Zealand Clinical Trials Registry (ANZCTR) https://www.anzctr.org.au/Trial/Registration/TrialReview.aspx?ACTRN=12620001003965
Protocol version {3}	11/12/2020 Version 5.0
Funding {4}	Victorian Cancer Agency Grant CPSRG19011
Author details {5a}	*Shakira Milton*^*1,2*^*, Jennifer McIntosh*^*2,3*^*, Finlay Macrae*^*,4,5*^*, Patty Chondros*^*2*^*, Lyndal Trevena*^*6*^*, Mark Jenkins*^*7*^*, Fiona M. Walter*^*2,8*^*, Lucy Boyd*^*1,2*^*, Sibel Saya*^*1,2*^*, Napin Karnchanachari*^*1,2*^*, Kitty Novy*^*1,2*^*, Carmody Forbes*^*1,2*^*, Javiera Martinez Gutierrez*^*1,2,9*^*, Kate Broun*^*10*^*, Sara Whitburn*^*11*^*, Sarah McGill*^*12*^*, George Fishman*^*13*^*, Julie Marker*^*13*^*, Max Shub*^*13*^*,Jon Emery*^*1,2,8*^ **Author Affiliations** 1. Centre for Cancer Research, University of Melbourne, Melbourne, Australia 2. Department of General Practice, University of Melbourne, Melbourne, Australia 3. Department of Software Systems & Cybersecurity, Monash University, Melbourne, Australia 4. Colorectal Medicine and Genetics, The Royal Melbourne Hospital, Melbourne, Australia 5. Department of Medicine, The University of Melbourne, Melbourne, Australia 6. School of Public Health, The University of Sydney, Sydney Australia 7. Melbourne School of Population and Global Health, University of Melbourne, Melbourne, Australia 8. The Primary Care Unit, University of Cambridge, Cambridge, United Kingdom 9. Department of Family Medicine, School of Medicine. Pontificia Universidad Católica de Chile, Chile 10. Early Detection and Immunisation, Prevention Department, Cancer Council Victoria, Australia 11. Belmore Road Medical Clinic, Melbourne, Australia 12. Cancer Screening and Prevention, Cancer Institute NSW, Australia 13. PC4 Joint Community Advisory Group, University of Melbourne, Australia JE conceived the study and led the initial study design. SM, JM, FM, PC, LT, MJ, FMW, NT, LB, SS, NK, KN, CF, JMG, KB, SW, SM, GF, JM, MS and JE contributed to the study design. JE, MJ, LT, FW, FM, JM, SS, PC, and SM are the grant holders. PC provided statistical expertise in clinical trial design. All authors contributed to refinement of the study protocol and approved the final manuscript.
Name and contact information for the trial sponsor {5b}	The University of Melbourne is the trial sponsor. Phone: 13 MELB (13 6352) International: +(61 3) 9035 5511 Postal address**:** The University of Melbourne, Victoria 3010 Australia
Role of sponsor {5c}	The sponsor and funder do not have ultimate authority over the study design; collection, management, analysis, and interpretation of data; writing of the report; and the decision to submit the report for publication.

## Introduction

### Background and rationale {6a}

In 2017, Cancer Council Australia published new guidelines recommending that people aged 50–70 years actively consider taking daily low dose aspirin to prevent colorectal cancer (CRC) [1]. This was based on a systematic review of the evidence and pooled data from four aspirin cardiovascular prevention trials which demonstrated a 25% reduced relative incidence and 33% reduced relative mortality from CRC with a median follow-up of 18.3 years [2]. The trials demonstrated the benefits of aspirin were seen with low dosages which varied from 75 mg to 300 mg per day. There is some evidence from sub-group analyses of the pooled data that taking aspirin for only 2.5–5 years may be as beneficial as consumption for more than 5 years [2]. The effects of aspirin on incidence and mortality appear to be stronger for the proximal colon [2], which is important as these tumours tend to present later and are more likely to be missed at colonoscopy [1]. People at increased risk of CRC, either due to their family history, or those with a history of adenomas, are likely to have a greater absolute benefit than those at average risk of CRC [3, 4].

There are some limitations to the evidence. Firstly, the aspirin cardiovascular preventions trials were designed primarily to assess the effect of aspirin on cardiovascular outcomes rather than cancer. Secondly, except for the Women’s Health Study (WHS), most participants in these trials were men. In the WHS the effect on reduced CRC incidence was observed but in modelling studies, the cardiovascular benefit dominated the reduction in CRC incidence [5]. Also, more recently, findings from the ASPREE trial did not show any benefit of low-dose aspirin in people aged over 70 in terms of all-cause mortality or disability-free survival [6, 7]. The ASPREE trial is not directly comparable as it was conducted in an older cohort, and unlike the other studies, the reported follow-up was only after a median of 4.7 years’ and therefore too soon to expect any effect on cancer incidence or mortality.

There is also evidence that aspirin is effective in reducing the risk for all cancers, not just CRC [8]. A meta-analysis of individual-level patient data from seven cardiovascular prevention trials demonstrated a 33% relative reduction in all cancer mortality after 5 years follow-up, an effect which persisted to 20 years; the effect was greatest for gastrointestinal cancers, with a 35% relative reduction in mortality within 20 years [8]. The cancer-preventive effects of aspirin are in addition to the established benefits in reducing cardiovascular disease (myocardial infarcts, ischaemic strokes and transient ischaemic attacks [9]. Aspirin also has well-recognised side effects including upper gastrointestinal symptoms and increased risk of haemorrhagic stroke [10–12]. However, fatal gastrointestinal bleeding rates did not differ between aspirin and placebo groups in pooled analyses [13]. Overall, the modelled benefits substantially outweigh potential harms from aspirin [14]. For example, it is estimated that, for a 50-year-old, taking aspirin for 10 years is 10 times more likely to prevent death than cause it, and five times more likely for someone aged 65 years. One death would be prevented for every 106 men aged 50 and for every 46 men aged 65 years by taking aspirin for 10 years [14]. This evidence has not yet been considered by Cancer Council Australia to inform guidelines about preventing other cancers.

We have developed expected frequency trees (EFTs) for an Australian population of 10,000 men or women aged 50–70 which present likely outcomes over ten years of taking aspirin for at least five years [14]. EFTs are graphical summaries that aim to simplify multiple conditional probabilities and present the likelihood of specific outcomes [15]. In our previous vignette study of 304 patients aged 50–70 in Victorian general practice, these EFTs were found to be easily understood by patients and preferred to icon arrays as a method of communicating the benefits and potential harms of taking aspirin, particularly because the numbers needed to be conveyed are small and difficult to display in an icon array. In this study, over 70% of participants said they would take aspirin based on the risk information presented in the EFTs [16].

In the subsequent I-MAGIC project we obtained further feedback about the EFTs and explored approaches to implementing the aspirin guidelines with relevant clinical groups. GPs, gastroenterologists, geneticists and pharmacists recognised a potential role for themselves in recommending aspirin, but there was consensus that general practice holds the key to widespread implementation of the guidelines [17]. This would form part of a larger implementation and communication strategy in the future.

The Cancer Council Australia guidelines recognise that the decision to take aspirin is a personal one, which accounts for a person’s disease risk but also their personal preferences and perceptions of the relative benefits and harms of taking daily aspirin [18]. Decision aids are an effective strategy for integrating research evidence with patient values, to allow shared decision-making. A Cochrane systematic review of 105 studies has shown that decision aids facilitate greater patient involvement, improve knowledge and increase value congruent choices [19]. Decision aids also improve the accuracy of patients’ risk perception and are particularly useful when decisions require weighing up of benefits and risks which may be preference-sensitive. Taking low dose aspirin exemplifies such a decision. There are no published trials of decision aids relating to taking aspirin to prevent cancer.

### Objectives {7}

This SITA trial aims to test the efficacy of a health consultation and use of decision aid, using an EFT to present the benefits and harms of taking low dose aspirin, on informed decision-making and use of aspirin in general practice.

The two equally important objectives are to determine if the novel-EFT based decision aid, used in a health consultation compared with general CRC prevention information:

1. Increases self-reported use of aspirin at 6 months amongst general practice patients between 50 and 70 years old, and
2. Increases informed decision-making related to taking aspirin at 1 month in general practice patients between 50 and 70 years old

Secondary objectives are to compare the novel EFT-based decision aid, used in a health consultation compared with general CRC prevention information in general practice patients between 50 and 70 years old with respect to:

1. Self-reported use of aspirin at 1 month
2. Lower mean decisional conflict at 1 month
3. Self-reported changes in other behaviours to reduce risk of CRC (e.g. dietary, quitting smoking, or having a screening test for CRC).

#### Primary hypotheses

1. An EFT-based decision aid, used in a health consultation, will increase regular use of aspirin for patients between 50 and 70 years old at 6 months compared with general CRC prevention information.
2. An EFT-based decision aid, used in a consultation, will increase informed decision making about aspirin use for patients between 50 and 70 years old after 1 month compared with general CRC prevention information.

### Trial design {8}

Multi-site, phase II single-blinded randomised controlled trial with 1:1 allocation conducted in 5–7 general practices in Victoria, Australia, with recruitment starting in October 2020 and final follow-up to occur by the end of 2021.

#### Methodological frameworks

We have applied the UK Medical Research Council Framework for the Evaluation of Complex Interventions to inform our pre-trial research, working with end-users to optimise the intervention [20]. This is a phase II efficacy trial which is more appropriate for novel interventions to test if an intervention, delivered in an ‘ideal way’, could work [21].

## Methods: Participants, interventions, and outcomes

The Victorian Cancer Agency (VCA) grant for this trial was granted in December 2019. The COVID-19 virus was first detected in Australia in January 2020 and by 13 March there were 140 confirmed cases which prompted the beginning of a lockdown nationally a few weeks later [22]. The State of Victoria has been the most heavily affected when compared to the rest of Australia and during the second wave of COVID-19 many people contracted it while at work [23]. As of 15 September, Victoria, primarily metropolitan Melbourne, where this trial was set, recorded 19,911 out of 26,738 total cases of coronavirus in Australia [24].

COVID-19 mitigation efforts in Victoria have included varying degrees of self-isolation, only leaving home for daily exercise, essentials and medical care, limited travel, border closures and an evening curfew [25]. In response to the lockdown there has been a 30% decline in face-to-face GP visits and an increase in telehealth consultations [26, 27]. To limit community transmission of COVID-19, from 13 March 2020, new temporary Medicare Benefits Schedule telehealth items were made available to help provide protection for patients and health care providers [28]. As COVID-19 has changed clinic waiting rooms and the number of people visiting their GPs face to face, we developed a teletrial method to deliver the trial virtually. The teletrial methods are explained below.

### Study setting {9}

This study is being conducted in three to six metropolitan and one to two rural general practices in Victoria, Australia.

### Sample size {14}

Preliminary unpublished data from the OPTIMISE trial [29] were used to inform sample size calculations for the two co-primary outcomes. In this general practice trial of an electronic multi-criteria decision analysis tool, 19% of 1780 participants aged 50–70 years were taking daily low dose aspirin at baseline; 34% of 38 participants in the control arm made an informed choice related to taking aspirin at 3 months. Based on these findings, for our trial, we assumed uptake of daily low dose aspirin at 6 months in the control arm will be 19%, given that those already taking aspirin will be excluded from this trial, and 34% of control participants will have made an informed choice at 1 month.

For 80% power and a Bonferroni adjusted 2-sided alpha level of 2.5% to account for the two co-primary outcomes [29], we require 258 participants (129 per arm) to detect a minimum 20% difference, as decided on by the trial steering committee because there is no current evidence for a minimal clinically important difference, between intervention and control arms in the (a) proportion of participants uptake of regular aspirin use at 6 months (39% vs 19%), and (b) proportion making an informed choice about aspirin use at 1 month (54% vs 34%). Sample size has been inflated to allow for 10% attrition after 1 month and up to 15% attrition after 6 months.

### Eligibility criteria {10}

#### Inclusion criteria for participants

Participants will be eligible if they are (i) aged between 50 and 70 years old and have an appointment with their GP on the day of recruitment or on the following day (ii) able to read and understand written English, and (iii) competent to give informed consent.

#### Exclusion criteria for participants

Patients will be ineligible if they (i) have contraindications to aspirin (e.g. previous peptic ulcer, taking anticoagulants); (ii) are already using aspirin regularly; (iii) are unavailable over the next 6 months to complete 1-month and 6-month follow-up questionnaires; (iv) have a previous diagnosis of bowel, endometrial, ovarian or stomach cancer; (v) there is family member with a known genetic variant associated with Lynch syndrome; or (vi) they meet any of the following high-risk family history criteria [30, 31]:

1. Three or more first- or second-degree relatives on the same side of the family diagnosed with CRC or other Lynch syndrome-related cancers (endometrial, ovarian, gastric, pancreatic, urothelial, renal pelvic, small intestine, biliary tract, brain).
2. Two or more first- or second-degree relatives on the same side of the family diagnosed with CRC including any of the following high-risk features: multiple CRC diagnoses in the one person, a CRC diagnosed younger than 50 years old, or a family member with a Lynch syndrome-related cancer.

#### Drop out or withdrawal criteria

The trial will be stopped for intervention or control group participants if they refuse to participate or continue with the follow-up questionnaires for any reason without explanation. The participants will have the choice to withdraw all their data if they withdraw before their data are analysed.

#### Inclusion criteria for clinics

General practices will be included if they have 3 or more full-time equivalent GPs to ensure a sufficient volume of potential participants.

#### Exclusion criteria for clinics

General practices are excluded if they have dedicated COVID-19 testing facilities with a high volume of symptomatic patients. This is to reduce the risk of viral exposure to research staff.

### Recruitment {15}

**Fig. 1:**
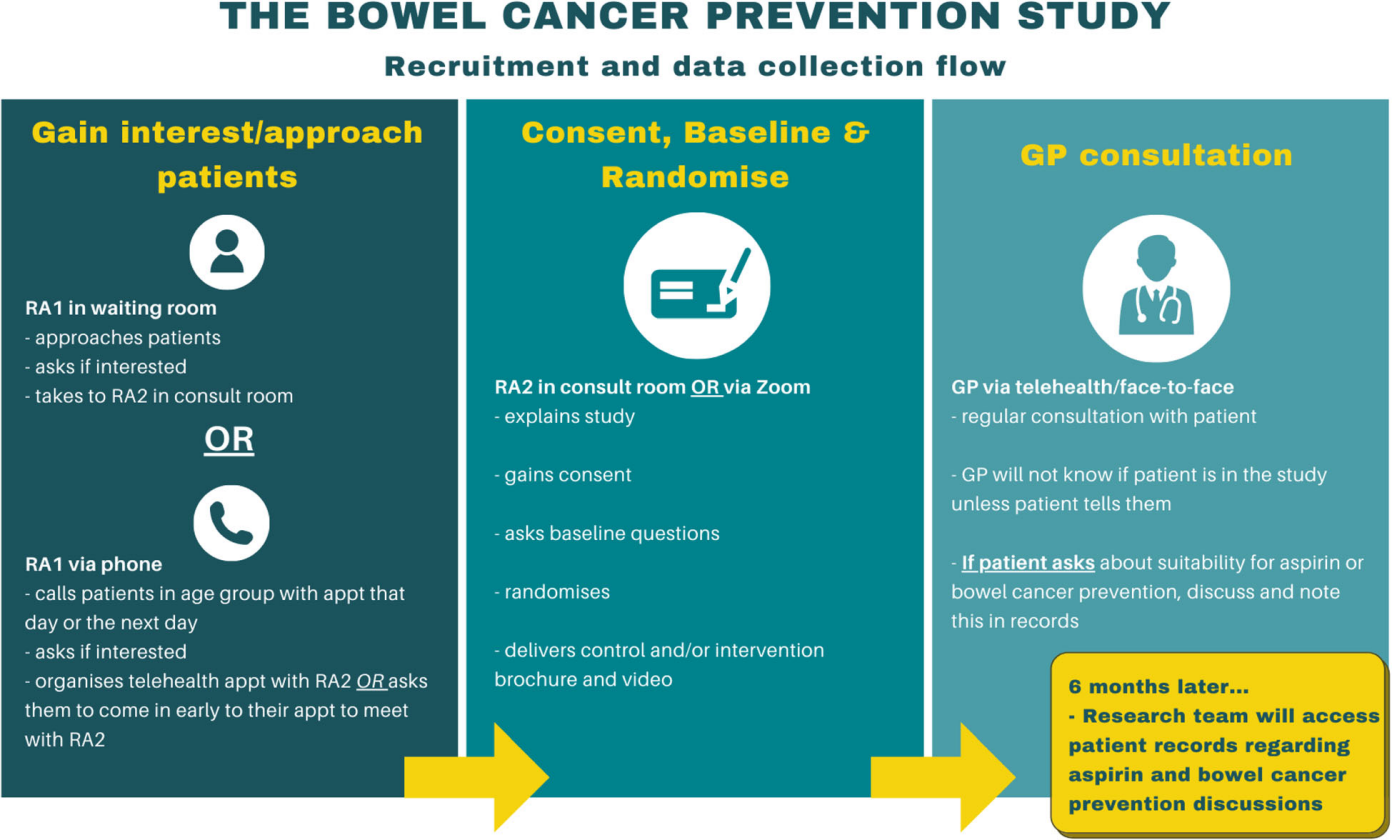
Overview of SITA trial recruitment and data collection flow

A visual overview of the trial recruitment can be found in Fig. 1. Due to the impact of COVID-19 on patterns of consulting in general practice, this trial has been adapted to include teletrial recruitment and delivery of the intervention and control in addition to standard face-to-face methods. During recruitment, two research assistants (RA1 and RA2) work together in a general practice. RA1 will be involved in the initial approach of the patients of the trial and RA2 will deliver the interventions after participants are randomised.

#### Face-to-face approach

When in a general practice, the recruiting staff will work closely with the practice administrative staff to identify potentially eligible participants who have an in-clinic appointment with their GP. RA1 will send a text message in advance of the phone call before calling patients who have an appointment on the day or the following day. If potential participants are interested and eligible, RA1 will schedule them in with RA2 for an appointment at least 15 min prior to their GP appointment. RA1 will also approach patients in the general practice waiting room and invite them to take part in the trial if they have not already been approached by RA1 on the phone. If the participant is eligible and interested in participating in the trial, they will be directed to RA2 in a private consulting room to obtain written informed consent and enrol in the study. To ensure there is minimal impact to general practice workflow, only participants whose GPs are running late by at least 15 min or who have arrived early for their appointment will be approached in the waiting room. We have successfully applied similar recruitment methods for a similar target population in the Colorectal RISk Prediction tool (CRISP) trial [32]. This method has been used effectively to maximise accrual and reduce recruitment bias [33].

#### Teletrial approach

Based on current data during the Victorian COVID-19 outbreak, approximately 30% of GP consultations will be delivered via a telehealth model [34]. In addition to the face-to-face waiting room method to invite patients into this trial, we have developed a teletrial approach both to recruit patients and deliver the trial. Recruiting staff will work with the administrative staff to obtain patient appointment lists to identify potentially eligible participants who have an appointment with their GP on the day or on the following day. Patients in the target age group will receive a text message in advance of the phone call, warning them they will be contacted to discuss a research project being conducted at their general practice. If the patient is eligible, the researcher will email or send to their mobile phone the Zoom Videoconferencing (Zoom) [35] link and the trial participant information brochure.

Depending on whether the participant has a teletrial or face-to-face appointment with their GP, delivery of the trial consultations will occur either via a password-protected zoom meeting or at the general practice before the participant’s GP appointment (see the ‘Interventions’ section)

### Who will take informed consent? {26a}

#### General practitioner informed consent (to allow research staff to recruit patients in their clinics)

Members of the research team will introduce the trial to all clinic staff in either a virtual meeting using Zoom or in-person via a PowerPoint presentation and invite discussion about the study (Fig. 1). This includes information about the Cancer Council Australia guidelines relating to aspirin to reduce the risk of CRC. Each GP will be given a participant information brochure, before obtaining written informed consent from GPs willing to participate, on the spot or prior to first patient recruitment. General practices will be recruited via a meeting with RA1 and RA2 and the individual GPs will provide written consent if interested.

#### Patient informed consent (to test the intervention)

Trained RAs will approach patients either face-to-face, in the practice waiting room or by telephone in advance of a booked appointment with their GP. For the full recruitment strategy, please see item in the ‘Recruitment {15}’ section.

Eligible patients will receive a participant information brochure either in person or via a Portable Document Format (PDF) attached to a welcome email after a phone call. After receiving the participant information, the patient and RA will have an open discussion where potential participants can ask questions about the trial.

Trained RAs will obtain informed consent from participants in the trial. For teletrial participants, electronic consent is captured via the study’s REDCap database.

For face-to-face recruitment, participants will sign a paper consent or complete the electronic consent form. See supplementary file A and B for GP and patient consent documents.

### Additional consent provisions for collection and use of participant data and biological specimens {26b}

Consent obtained in this study is specific and will not be used for future projects. Biological specimens will not be collected as part of this study.

## Interventions

### Intervention description {11a}

**Fig. 2:**
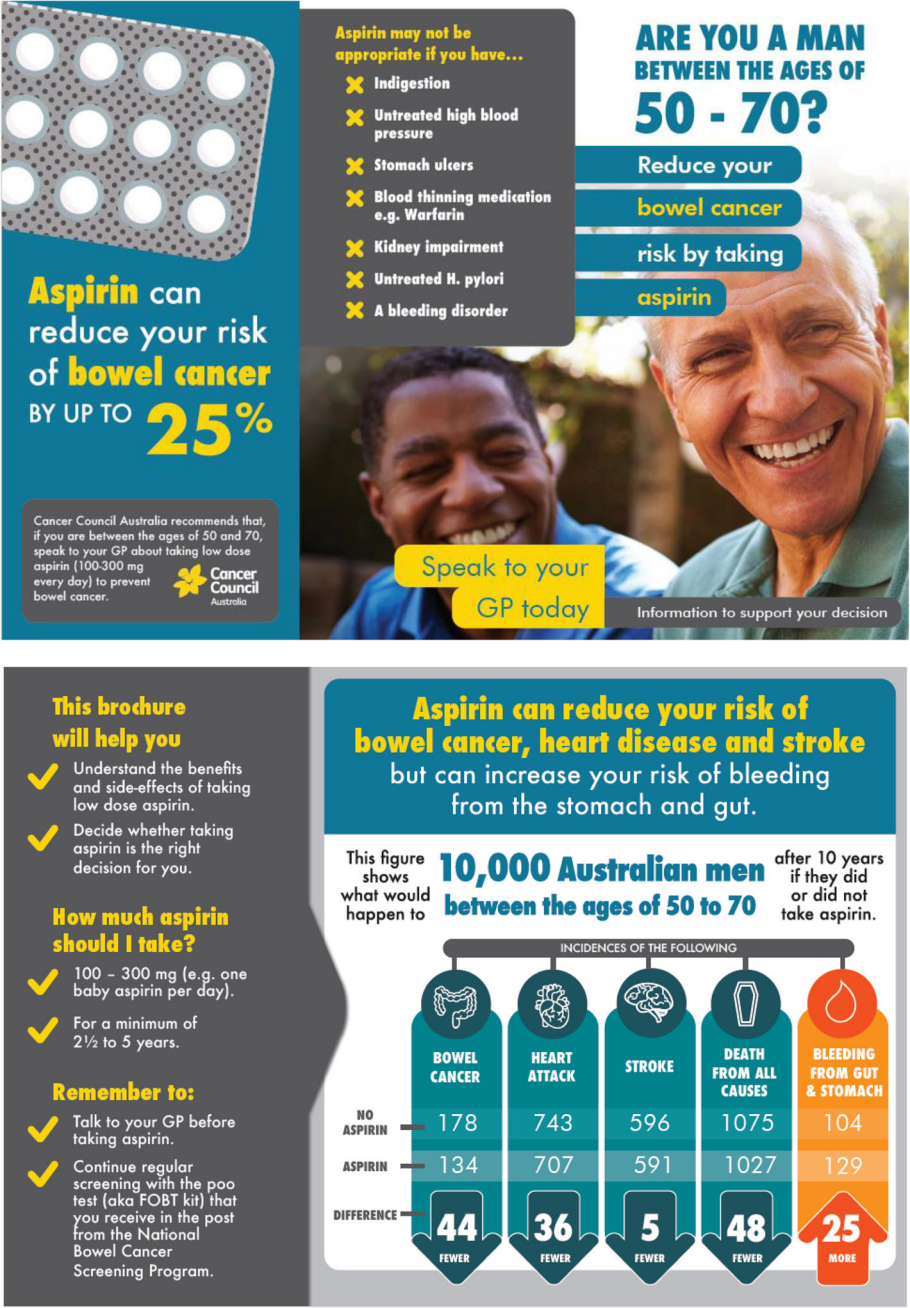
Tri-fold male decision aid which communicates the harms and benefits of taking aspirin for CRC prevention

**Fig. 3:**
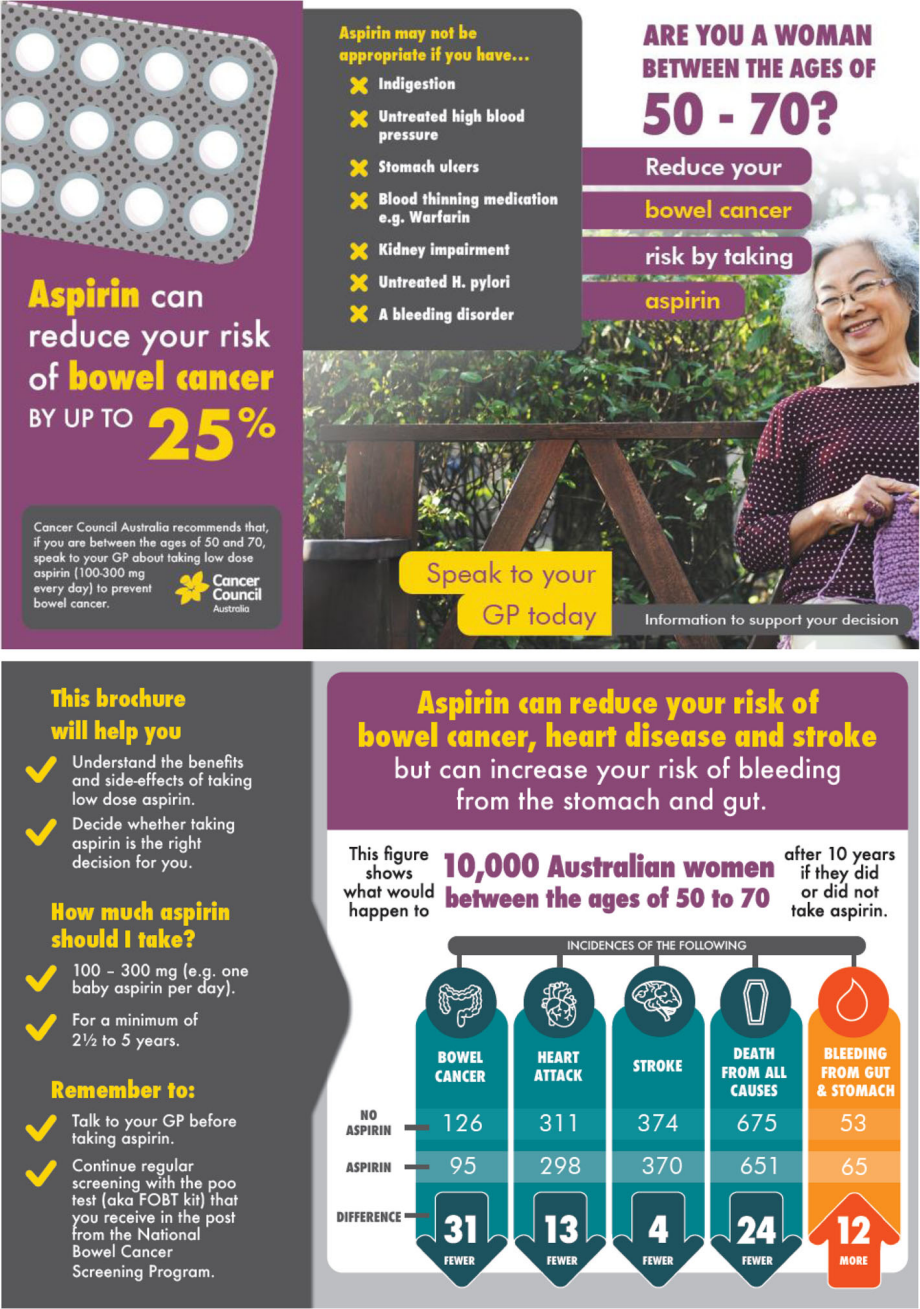
Tri-fold female decision aid which communicates the harms and benefits of taking aspirin for CRC prevention

Participants in the intervention arm will attend a health consultation either in person or by Zoom, delivered by a trained researcher, during which a decision aid for females or males (Figs. 2 and 3) about taking aspirin to reduce risk of CRC and other chronic conditions will be discussed. Initially, their risk of CRC and cardiovascular disease will be assessed qualitatively based on family history of CRC and self-reported cardiovascular disease risk factors collected in the baseline questionnaire. To determine participants’ cardiovascular disease risk, RA2 will ask four questions at baseline (see baseline questionnaire, in the Supplementary file C). If the participants answer yes to either of the questions, they will be told that the size of the potential benefit of using aspirin could be greater than presented in the decision aid and they should ask their GP about aspirin in relation to their increased cardiovascular disease risk as well as for CRC prevention.

Using a standardised consultation script, the decision aid will be discussed in the context of any CRC and cardiovascular disease risks and personal values and preferences, allowing the participant to decide if taking aspirin is the right decision for them.

A brief video of the decision aid will be used for both face-to-face and teletrial delivery of the intervention. A copy of the decision aid will be provided to intervention participants after the video is discussed and viewed. Teletrial participants will receive an email immediately following the consultation with a PDF two-page version of the decision aid and a hard copy will be posted to them.

The decision aid is a two-page tri-fold colour brochure. It includes the following information presented in plain language: a statement of the Cancer Council Australia recommendation; potential contraindications to using aspirin; advice about dose and duration of taking aspirin; advice to discuss whether to take aspirin with their GP; an EFT which presents absolute risks of CRC, ischaemic heart disease, stroke, all cause deaths and gastrointestinal bleeding in a population of 10,000 Australian men or women. Please see supplementary file F for the two-page version of the decision aid which were included in emails to teletrial participants, file G, and file H for the video decision aids for females and males, respectively.

Intervention participants also receive the control arm brochure outlining other ways to prevent CRC. They will be encouraged to discuss aspirin use with their GP before commencing it and will receive an SMS reminder two weeks after enrolment to discuss aspirin with their GP if they have not done so already.

### Explanation for the choice of comparators {6b}

**Fig. 4:**
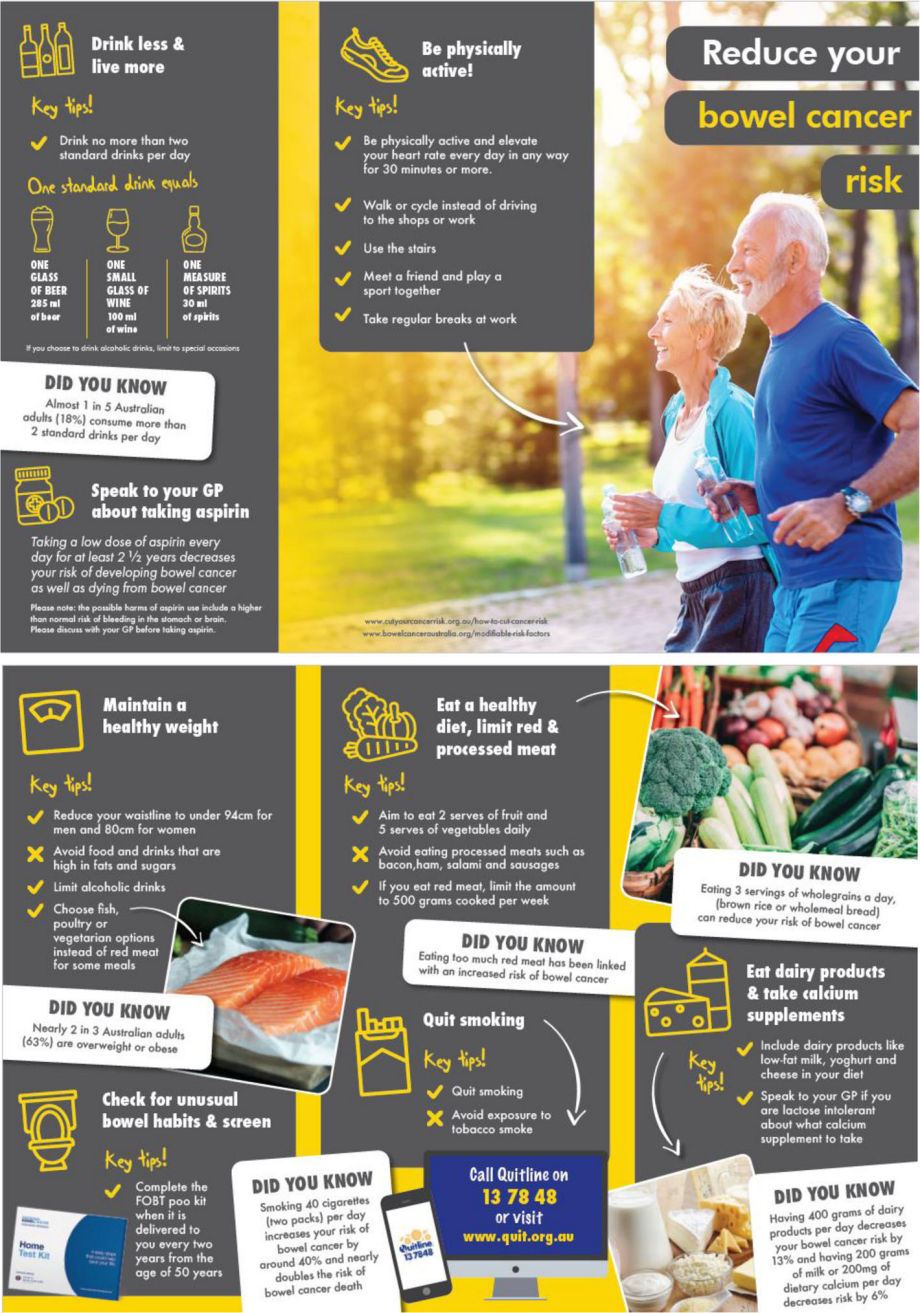
Tri-fold brochure for the control and intervention participants in the SITA trial, which includes advice on how to reduce the risk of CRC

Control arm participants will receive, general information about CRC prevention. The same trained researcher, RA2 will use a standardised consultation script to discuss a brochure (Fig. 4) and video that covers up-to-date information about CRC prevention and screening from Cancer Council Victoria and Bowel Cancer Australia including maintaining a healthy weight, a reminder to continue CRC screening, quitting smoking, eating dairy products, taking calcium supplements, drinking less alcohol, healthy diet, being physically active, and speaking to their GP about taking aspirin [30].

The video format for the brochure was created so that all participants regardless of the mode of delivery (teletrial or face-to-face) receive the information in the same format and order. A copy of the brochure will be provided to all participants after the video is discussed and viewed. All teletrial participants will receive an email immediately following the consultation with a PDF two-page version of the control brochure and a hard copy will be posted to them. See Supplementary file D for the control video and file E for the control email brochure.

We will audio record 10% of the trial consultations to ensure fidelity in delivering both intervention and the comparator consultation scripts.

### Criteria for discontinuing or modifying allocated interventions {11b}

Participants can withdraw from the trial at any time, without providing reason. They may elect to not be contacted again or they may withdraw all their data from the trial, but they will not be able to withdraw data once it has been deidentified and aggregated.

If the RA runs out of time and participants are called into their GP appointment before they consent or before randomisation, they will not be included in the trial. If the RA runs out of time after a participant is randomised, we will give the participant the decision aid and/or control brochure and they will be included within the intention-to-treat analyses.

Study arm allocation is random, participants cannot choose which arm they are in. Further, participants are not notified of their study arm allocation, they are given either one brochure or two brochures, it is therefore unlikely a participant will know which arm they have been allocated.

### Strategies to improve adherence to interventions {11c}

Before commencing the trial in a general practice, we will present the Cancer Council Australia guidelines and contraindications to aspirin with each GP as a part of the consenting process (see {26a}). As GPs are commonly unaware of these guidelines [17], we will use this discussion and the decision aid to raise awareness of the new recommendations about aspirin. To reduce the risk of contamination in the control arm, we will encourage each GP to continue their normal practice and only to respond to patients’ queries about aspirin.

Participants in the intervention arm will be encouraged to speak with their GP about aspirin for CRC prevention before commencing it. We will assess the potential degree of contamination between trial arms by measuring GP discussions about aspirin in the 6 months after randomisation through the audit of GP medical records at 6 months.

### Relevant concomitant care permitted or prohibited during the trial {11d}

N/A

### Provisions for post-trial care {30}

We do not anticipate any harm to participants during or after the trial arising directly from the decision aid. To ensure participant concerns, following participation in the trial are adequately managed they participate just before their scheduled GP appointments and are urged to speak to their GP about any concerns they may have.

### Outcomes {12}

Outcomes will be measured at 1 and 6 months.

Two co-primary outcomes will be assessed for the trial.

1. The difference between the two-study arms in the proportion who self-report regular adherence to daily aspirin (i.e. taken 5 or more out of 7 days in a week) at 6 months.
2. The difference between the two-study arms in the proportion who have made an informed decision about taking aspirin at 1-month measured using the Multidimensional Measure of Informed Choice (MMIC) [36].

Secondary outcomes include the between-arm difference in:

1. Proportion who self-reported regular adherence to daily aspirin (i.e. taken 5 or more out of 7 days in a week) at 1 month.
2. Mean Decisional Conflict score at 1 month measured using the Decisional Conflict Scale (DCS) [37]
3. Proportion who prefer to take aspirin to reduce chances of bowel cancer at 1 month
4. Proportion of participants taking aspirin to reduce the risk of CRC compared to those taking it to reduce the risk of cardiovascular disease and stroke
5. Proportion of self-reported changes to the each of the following behaviours to reduce risk of CRC at 1 month and 6 months:
  Changed diet
  Talking to their GP about quitting smoking
  Quit smoking
  Talked to GP about completing a bowel cancer screening test
  Completed a bowel cancer screening test (FOBT)
  Talked to GP about colonoscopy
  Talked to GP about having a colonoscopy
  Talked to their GP about taking aspirin.

Please see supplementary files C, J and K for the baseline, 1-month and 6-month follow-up questionnaires.

### Participant timeline {13}

Table 1 shows the participant timeline.

**Table 1:** Participant timeline

	Recruitment	Enrolment	Allocation	Post-allocation		
Timepoint**	-t_**2**_	-t_**1**_	0	0 months	1 month	6 months
**Enrolment:**						
**Eligibility screen**	X					
**Baseline questionnaire**		X				
**Informed consent**		X				
**Allocation**			X			
**Interventions:**						
**Aspirin decision aid**				X		
**General CRC prevention brochure**				X		
**Assessments:**						
**Subjective numeracy scale**		X				
**Decisional conflict**					X	
**Multidimensional measure of informed choice**					X	
**Self-reported daily adherence to aspirin**						X
**Discussion about CRC prevention with GP (GP records)**						X

## Assignment of interventions: allocation

### Sequence generation {16a}

Participants will be randomly allocated 1:1 to the intervention or control arm. The allocation sequence will be computer-generated by the study statistician, stratified by general practice, brochure type based on sex (male or female) and mode of trial delivery (face-to-face or teletrial) using permuted blocks of random sizes. To ensure concealment the block sizes will not be disclosed to the recruiting staff or investigators. Participants who did not identify as either male or female, or tick ‘other’ as their option for sex in the baseline questionnaire, will be asked, if they had a choice between two brochures designed for male and females which brochure they would prefer (male or female version). Based on their brochure preference, they will be allocated to either the male or female stratum.

### Concealment mechanism {16b}

The allocation schedule will be embedded within the online database, REDCap, which will automatically assign the participant after they complete the baseline survey to either intervention or control arm ensuring allocation concealment.

## Assignment of interventions: Blinding

### Who will be blinded {17a}

Participants will be told that they are participating in a trial which aims to test information brochures about ways to reduce their risk of CRC. They will be blinded to the study arm allocation. The RAs will not disclose how the information presented in the brochures differs. Outcomes assessed by self-report obviate the need for researcher blinding. A separate RA who is not involved in the participant recruitment will be responsible for ensuring follow-up of 1- and 6-month questionnaire responses will also be blinded to study arm allocation. Due to the nature of the study, GPs will not be blinded to the study arm allocation as the participants in the intervention arm are advised to speak to their GP about taking aspirin. GPs will be provided with a copy of each decision aid and the control brochures but will be advised to discuss aspirin or other ways to reduce CRC risk only if their patients specifically raise the issue.

GP electronic medical records will be audited after 6 months to validate the self-reported adherence to aspirin by RA1, who will be blinded to the participant allocation.

### Procedure for unblinding if needed {17b}

We do not anticipate the need to unblind the trial. The only potential exception would be if a participant experienced a serious health event which could potentially be related to the use of aspirin. In such a circumstance, the RA who allocated the participant and delivered the intervention will be our first point of contact. The University of Melbourne Human Research Ethics Committee would be contacted and the investigators including all members on the trial steering committee would be notified.

## Data collection and management

### Plans for assessment and collection of outcomes {18a}

Participants will complete a baseline questionnaire which will be administered by the RA2 prior to randomisation and entered directly into the RedCap trial database, in a private consultation room. One and 6-month follow up a link to the online questionnaires for the patient-reported outcome measures will be sent to each participant by either text, email, over the phone by an RA who is not involved in recruitment or by receiving a paper copy in the post depending on their stated preference at baseline. Participants who receive the follow-up questionnaire by text and email will receive two automated text or email reminders to complete the questionnaires after 3 and 6 days and will receive a phone call reminder by a blinded RA after 9 days. Participants who receive the follow-up questionnaires by post will be reminded by phone to return them 10 days after they are posted. If the participant does not have a phone number, we will repost the questionnaire, with a reminder note attached, after 2 weeks if we do not receive back the first one.

Participant demographic characteristics collected at baseline will include date of birth, sex (male, female or other), postcode of residence, education level (never completed high school, completed high school only, TAFE qualification or similar, or University degree or higher), country of birth, number of current medications taken daily, living arrangements (living alone or with others) and language spoken at home.

The validated 8-item Subjective Numeracy Scale [38] will be administered at baseline to assess participants’ comprehension and preferences for numerical information including probabilities, proportions, and percentages. Four items measure people’s beliefs about their skill in performing various mathematical operations, and four items measure their preferences regarding the presentation of numerical information. Response values increase left to right (1–6) for all the items, except Question 7 which is reverse coded (6–1). The scale is calculated by taking an average across the 8-item items, with higher scores reflecting higher subjective numeracy.

Participants’ cardiovascular disease risk factors will be self-reported by answering the following questions (yes, no or unsure): a family history of heart attack, angina, or stroke; a personal history of diabetes; medication for high blood pressure; personal history of high cholesterol; and a personal history of smoking cigarettes. Similarly, participants’ CRC familial risk will be self-reported by answering the following (yes, no or unsure): a family history of CRC (parent, brother, sister, children) diagnosed before 55 years old, and more than one relative who had CRC at any age (parents, children, brothers, sister, grandparents, aunts, uncles, nieces, nephews and grandchildren).

#### Primary outcomes

1. Self-reported regular adherence to daily aspirin (i.e. taken 5 or more out of 7 days in a week) at 6 months as a yes/no response. Participants who respond “not taken aspirin in the last month” or “started and then stopped” will be coded as not having adhered to daily aspirin use [39].
2. Informed choice to take aspirin will be measured using MMIC, which is a composite measure of knowledge, attitudes and behaviour [36], and has been used in several studies of cancer screening and genetic testing [37, 38]. We will use a set of aspirin-specific knowledge items developed for the OPTIMISE trial, for which a score of eight or more constitutes adequate knowledge (maximum score of 11) [29]. An informed choice is one where a participant has adequate knowledge and their behaviour (i.e.. to take aspirin or not) is consistent with their attitudes towards that behaviour (e.g. positive or negative attitudes towards using aspirin to prevent CRC). All other choices (i.e. with inadequate knowledge and/or a behaviour discordant with their attitude towards taking aspirin) are defined as uninformed. Attitudes towards the decision to take aspirin will be measured as reported in Marteau et al. [40] (minimum total score four, maximum 28, low scores indicating a more positive attitude, high scores a more negative attitude). We will use the mid-point of the scale to classify positive and negative attitudes (scores ranging from 12 to 20).

#### Secondary outcomes

1. The Decisional Conflict scale has 16 items, with three sub-domains (1) participants’ uncertainty about making a health-related decision, (2) factors that contribute to uncertainty, and (3) participants’ perception of how well they came to their final decision [37]. The Decisional Conflict score (range from 0 to 100), is calculated as the average of the 16 items scored on a Likert scale (0 = strongly disagree, 1 = agree, 2 = neither, 3 = disagree and 4 = strongly agree) and multiplied by 25, where 0 indicates no decisional conflict and 100 indicates an extremely high decisional conflict. The DCS has been widely used in the evaluation of decision aids [41]. The inverse correlation was (r 0.16, *p* < 0.05) between the decisional conflict scale and knowledge test scores showing the validity and the test’s acceptability [42].
2. Additional behaviours to reduce risk of CRC. At 1 and 6 months participants will be asked whether they have done any of the following things to reduce their chances of getting bowel cancer since they joined the study including making changes to their diet, talking to their GP about quitting smoking, quitting smoking, discussed with their GP screening for CRC by faecal occult blood test (FOBT) or colonoscopy, completed screening for CRC by FOBT or colonoscopy or talked to their GP about taking aspirin.
3. Self-reported regular adherence to daily aspirin (i.e. taken 5 or more out of 7 days in a week) at 1 month using the same measure as for the primary outcome at 6 months.
4. GP medical records will be audited to identify the proportion of participants who had a consultation in which aspirin use was discussed.

#### Additional descriptive measures

At 1 month, participants will be asked their preference out of four choices to reduce their risk of bowel cancer (change my diet, take aspirin, do the bowel cancer screening test or unsure).

Participants who answered “yes” or “started then stopped taking aspirin” to the questions about aspirin adherence will be asked additional information about the dose of aspirin they were taking (100 mg/300 mg/other); reasons for taking aspirin (reduce risk of heart attack, reduce risk of stroke, reduce my risk of bowel cancer), other (please specify); whether they experienced side-effects while taking aspirin (yes/no); and if yes, specific side-effects (participants will be provided with a list of the most common side-effects including nausea, easy bruising, indigestion, and bleeding). If they mention other side-effects that are not listed, they will be asked to describe them. At 6 months participants will also be asked the reasons why they did not take aspirin or why they stopped taking aspirin.

Please see Supplementary files C, J and K for the baseline, 1-month and 6-month follow-up questionnaires.

### Plans to promote participant retention and complete follow-up {18b}

Participants will receive a text message two weeks prior to being sent the 1-month and 6-month follow-up questionnaires. Participants will have the option to complete the questionnaires online (via email or text weblink), mail or administered over the phone with an RA. Reminder e-mails and/or phone calls will occur at pre-specified durations of non-response dependent on participants’ preferred method of follow-up.

Participants who do not complete the follow-up questionnaires will be included in the medical record audit 6-months after baseline, unless the participant actively withdraws consent to use their data.

### Data management {19}

Data will be collected on site in general practices and recorded directly in REDCap for all participants. The REDCap database will only be accessible by authorised university trial staff. REDCap is password protected with multi-factor authentication for additional security. The REDCap database has mandatory data entry fields to reduce missing data. Before randomisation, there is a check in REDCap to ensure accurate entry of the stratifying variables. All paper-based, will be entered directly into REDCap by an RA who was not involved in recruiting the participants, follow-up questionnaires will be stored securely in an office within the Victorian Comprehensive Cancer Centre in a locked file cabinet; all data will only be accessible to the listed researchers.

### Confidentiality {27}

Research data will be stored in accordance with the University of Melbourne’s Research Data Management Policy and Research Code of Conduct and will be stored on University managed and/or sanctioned storage infrastructure. Data will be secured via a personal login and data elements restricted by role at the direction of the Chief Investigator. After data collection, all identifiers such as participant names will be removed and replaced by a code. Electronic data will be re-identifiable for the duration of project. Participant contact information (phone number and email address) will be stored in a quarantined area on REDCap, only visible to members of the research team who require it for study-related contact. This restriction will be built into REDCap user roles. Personal identifiers will be removed at trial completion, and only non-identifiable data will be stored subsequently. Paper-based data will be destroyed using confidential waste management services five years after the publication of the results.

## Statistical methods

### Statistical methods for primary and secondary outcomes {20a}

Descriptive statistics will be used to compare baseline measures between the two study arms. These include participant demographic characteristics, subjective numeracy score, self-reported cardiovascular risk and family history of bowel cancer. All randomised participants will be included in the main analysis in their assigned study arms in accordance with the intention-to-treat principle [43].

The two co-primary outcomes, (1) proportion of participants who are taking regular aspirin at 6 months and (2) proportion of participants who make an informed choice about taking aspirin at 1 month, will each be compared between the two study arms using logistic regression with general practice, brochure type based on sex (male or female) and mode of trial delivery (face-to-face or teletrial) included as covariates.

We will also use logistic regression for the secondary binary outcomes and linear regression for the continuous outcomes, and all regression analyses will be adjusted for the randomisation stratification factors.

The estimated intervention effect will be reported as the odds ratio for binary outcomes and the difference in means between the intervention and control arms for continuous outcomes. Estimates for the co-primary outcomes will be reported with Bonferroni adjusted 95% confidence intervals and p values. Estimates for secondary outcomes will be reported with respective 95% confidence intervals and p values with no adjustments for multiplicity [44].

All analyses will be conducted using Stata 15 [45].

### Interim analyses {21b}

We do not plan to conduct an interim analysis for this trial.

### Methods for additional analyses (e.g. subgroup analyses) {20b}

A sensitivity analysis may be performed on the primary and secondary outcomes to adjust for additional pre-specified baseline variables in the regression models. These include age in years, sex and family history of colorectal cancer, cardiovascular disease risk and subjective numeracy scores.

Exploratory sub-group analyses are planned by face-to-face versus teletrial, brochure type (male/female), cardiovascular risk, family history of bowel cancer, number of medications, and Socio-Economic Indexes for Areas (SEIFA) based on participants’ postcode of residence [46].

### Methods in analysis to handle protocol non-adherence and any statistical methods to handle missing data {20c}

An adherence adjusted analysis will be conducted for the two co-primary outcomes using a complier average casual effect (CACE) analysis [47]. Multiple imputation may be used to handle the missing data if appropriate. A sensitivity analysis using pattern-mixture model will assess the robustness of the missing data assumption. A detailed analysis plan will be developed for the secondary and sensitivity analyses.

### Plans to give access to the full protocol, participant level-data and statistical code {31c}

Only the study team will have access to the participant-level dataset stored in REDCap. All the authors will have access to the full protocol.

The statistical code including allocation schedule will only be available to the trial statistician and PhD candidate.

## Oversight and monitoring

### Composition of the coordinating centre and trial steering committee {5d}

#### Principal investigators and PhD Candidate

Design and conduct of SITA

Decide when site clinics visits occur

Preparation of protocol and revisions

Organising steering committee meetings

Publication of study reports

Data management

Maintenance of trial data management system REDCap, and entry of study data

#### Steering committee (SC)

(see title page for members)

Agreement of final protocol

Reviewing progress of study and if necessary, agreeing changes to the protocol to facilitate the smooth running of the study.

#### Recruitment team including RAs

Study planning

Organisation of steering committee meetings

Provide annual risk report MHRA [Victorian Cancer Agency] and ethics committee

Recruitment of patients and liaising with the principle investigator

Budget administration and contractual issues with individual centres

Assistance with Ethics

Data verification

Randomisation

Delivery of intervention to intervention or control participants

### Composition of the data monitoring committee, its role and reporting structure {21a}

This trial is testing the efficacy of a decision aid; any final decisions to take aspirin will be left to the participant in discussion with their GP. The intervention itself therefore is relatively low risk. This is a relatively small phase II efficacy trial. We do not expect significant adverse effects arising from the trial itself. We have therefore decided not to have a separate data monitoring committee. Oversight of the trial will be managed by the trial steering committee.

### Adverse event reporting and harms {22}

This study aims to allow participants to make an informed choice about following a nationally recommended approach to reduce CRC risk: taking low dose aspirin. We recommend that participants who are considering taking aspirin discuss it with their GP to ensure it is safe for them. The potential risks of involvement therefore relate to those of decision-making and not of taking aspirin. These include low risk of anxiety in discussing CRC risk and uncertainty about options to reduce that risk. There is potential for participants to experience side effects from taking aspirin if they choose to take aspirin after discussion with their GP. All participants will be monitored by their GP if they commence taking aspirin and are advised to see their GP if concerns about potential side effects arise. We are collecting side-effects and adverse events from self-report and audit of the GP medical records.

### Frequency and plans for auditing trial conduct {23}

The recruitment team will meet weekly with the principal investigator (J Emery) to discuss the progress of the data collection and analysis and be on hand to manage any unforeseen situations including adverse events relating to aspirin use. The Trial Steering Committee of investigators named on the VCA grant will meet quarterly to discuss all aspects of the trial and progress. Overall progress will be reported to the VCA every 6 months*.* There will be no independent auditing of trial conduct.

### Plans for communicating important protocol amendments to relevant parties (e.g. trial participants, ethical committees) {25}

Any important protocol modifications will be discussed with the investigators and reported to the University of Melbourne’s Faculty of Medicine, Dentistry and Health Science’s Human Research Ethics Sub-Committee. Additionally, any modification in the protocol will also be updated in the ANZCTR. It is unlikely any significant changes necessitating participant communication will be made to the protocol, therefore there is no specific plan to communicate these changes. If needed, plans will be made to communicate these changes to participants accordingly.

### Dissemination plans {31a}

The results of this trial will be published in a peer-reviewed journal and reported at peer-reviewed scientific conferences and meetings.

The Primary Care Collaborative Cancer Clinical Trials Group (PC4) has a well-established communication strategy that would include the following: media releases to a health professional and general outlets; Twitter and other social media outlets; PC4 Research Round-up and other health professional and general podcasts; dissemination via the PC4 Consumer Advisory Group and their respective consumer networks. We would use all these approaches to promote the trial results and the decision aid. In addition, at the end of the trial we will hold a Think Tank involving key researchers, clinicians (e.g. GPs, gastroenterologists, practice nurses) and their representative colleges (RACGP, ACCRM, GESA, APNA), consumers and consumer organisations (e.g. Cancer Council Victoria, Bowel Cancer Australia), and health policy makers from the Victorian Department of Health and Human Services Prevention and Population Health Branch, Cancer Institute NSW, and Cancer Australia. We will specifically identify and invite representatives of rural GPs and consumers. At this event, we would present the key findings of the research and plan a range of strategies to promote the results and their uptake into practice. The Think Tank would be funded by PC4.

## Discussion

Aspirin can reduce the risk of developing CRC by up to 25% and the benefits outweigh the risks of taking it for most people aged 50–70 years [48]. Australia is the first country to have national guidelines recommending all people aged 50 to 70 consider low dose aspirin to prevent CRC, irrespective of other disease risks [13]. Decision aids are interventions with the potential to support informed choice by improving the following factors: knowledge, clarity of personal values, and implementation of an intention [49]. This trial is the first to develop and determine whether a decision aid is effective in increasing uptake and supporting an informed choice for patients aged 50–70 years about taking aspirin to prevent CRC and other common conditions.

We have developed a teletrial model in addition to previous waiting room methods (37) to administer the trial. By aiming to incorporate the teletrial methods we hope to capture general practice patients who have an appointment with their GP via telehealth who will not be visiting the practice. The uptake of telehealth consultations with GPs was approximately 30% between April and June 2020 in Australia as a result of COVID-19 [34]. Many non-COVID trials have been paused during this time which resulted in the need for an innovative approach to including study participants in this trial [50].

This trial will create new evidence on the efficacy of a decision aid about aspirin to reduce risk of CRC and CVD on aspirin use and informed decision-making. It will inform future models to implement Australian guidelines about using aspirin to prevent CRC.

## Trial status

The trial was approved by the University of Melbourne’s Medicine and Dentistry Human Ethics Sub-Committee on 28-Jul-2020.

This is version 5.0 of the protocol. The date of submission: 01/04/2021

The planned dates for recruitment are September 2020 and completion of recruitment August 2021.

Trial registration

The Australian New Zealand Clinical Trials Registry (ANZCTR) ACTRN12620001003965.

## Supplementary Information

**Additional file 1: File A**. Consent form for general practitioners. **File B**. Consent form for participants. **File C**. Baseline questionnaire. **File D**. Control video. **File E**. Control email brochure. **File F**. Teletrial decision aid for email. **File G**. Video decision aid for males. **File H**. Video decision aid for females. **Files I**. 1-month follow up questionnaire. **File J**. 6-month follow-up questionnaire

## Abbreviations

RA: Research assistant
EFT: Expected frequency trees
CRC: Colorectal cancer
GP: General practitioner
ANZCTR: Australia New Zealand Clinical Trial Registry
WHS: Women’s Health Study
VCA: Victorian Cancer Agency
