# Supplementary material for: An RCT of a decision aid to support informed choices about taking aspirin to prevent colorectal cancer and other chronic diseases: a study protocol for the SITA (Should I Take Aspirin?) trial
Source: Trials. 2021 Jul 15;22:452. doi: 10.1186/s13063-021-05365-8 (PMC8280579; doi:10.1186/s13063-021-05365-8)
Supplement: Supplementary file 1 — Additional file 1: File A. Consent form for general practitioners. File B. Consent form for participants. File C. Baseline questionnaire. File D. Control video. File E. Control email brochure. File F. Teletrial decision aid for email. File G. Video decision aid for males. File H. Video decision aid for females. Files I. 1-month follow up questionnaire. File J. 6-month follow-up questionnaire [file 13063_2021_5365_MOESM1_ESM.pdf]

ID: _ _ _ _ _ _ _
Date: _ _/_ _/_ _

Centre for Cancer Research & Department of General Practice University of Melbourne

CONSENT FORM FOR GENERAL PRACITIONERS

Project: The Bowel Cancer Prevention Study

Investigators:
Ms Shakira Milton, Prof Finlay Macrae, Prof Jon Emery on behalf of other SITA investigators

1. I consent to participate in this research project, the details of which have been explained to me, and I have been provided with a written participant information brochure (Participant PLS version 2.0, Date: 31/07/20) to keep.
2. I agree that the researchers may use the results as described in the participant information brochure.
3. I understand that after I sign and return this consent form it will be retained by the researcher.
4. I understand that my participation will involve discussing participants' risk of cancer, heart attack and stroke and bowel cancer prevention strategies.
5. I understand that my participation will involve discussing aspirin use to reduce the risk of developing colorectal cancer with participants aged 50 to 70 years (participants in the intervention group).
6. I understand my patients will be invited to be recruited prior to a consultation through being called a day prior to their appointment or approached in the clinic waiting room and that they will be consenting to allow the release of their medical information from my patient records. The only data that will be collected will relate to aspirin uptake and side effects for the purposes of this research only.
7. I understand that I might be contacted to be interviewed about my experiences of being involved in this trial, which will be audio recorded but only used for research purposes.
8. This project is funded by a grant from Victorian Cancer Agency (CPSRG19011).
9. I acknowledge that:
   a. The possible effects of participating in the research study have been explained to my satisfaction;
   b. I understand that my involvement in this study is entirely voluntary and will not affect my care in any way;

c. I have been informed that I am free to withdraw from the study at any time without explanation or prejudice and to withdraw any unprocessed data I have provided;

d. The study is for the purposes of research;

e. I have been informed that the confidentiality of the information I provide will be safeguarded subject to any legal requirements;

f. I have been informed that my consent form and information will be stored securely at the University of Melbourne and destroyed 5 years after publication of the study results;

g. I have been informed that a copy of the research findings will be forwarded to me, should I request it at the completion of the study;

h. In accordance with the law of Victoria, I understand that it is possible for data to be subject to subpoena, or freedom of information request.

11.	Contact from our GP Circle Network<br><br>We have set up a network for GPs who would like to help shape our research. You may then be contacted in the future about joining the research network.<br><br>☐ Yes, I would like to be contacted in the future<br>☐ No, I would not like to be contacted in the future

Name of participant: ____

Participant signature: _______________________________________ Date: ___________

ID: _ _ _ _ _ _ _
Date: _ _/_ _/_ _

Centre for Cancer Research & Department of General Practice University of Melbourne

CONSENT FORM FOR PARTICIPANTS

Project: The Bowel Cancer Prevention Study

Investigators:
Ms Shakira Milton, Prof Finlay Macrae, Prof Jon Emery on behalf of all SITA investigators

1. I consent to participate in this research project, the details of which have been explained to me, and I have been provided with a written participant information brochure (Participant PLS version 2.0, Date: 31/07/20) to keep.
2. I agree that the researchers may use the results as described in the participant information brochure.
3. I understand that after I sign this consent form it will be retained by the researcher.
4. I understand that the group I will be in is random and I cannot choose. I understand that my participation involves a short consultation with a researcher and receiving at least one type of information brochure about ways to reduce my risk of bowel cancer.
5. I understand that my participation will involve completing two questionnaires 1 and 6 months from today.
6. I agree to researchers obtaining information from my GP medical record about consultations relating to bowel cancer prevention in the next 6 months.
7. I understand my research consultation may be audio-recorded and that this is voluntary. The recording will only be used for quality assurance purposes.
8. I understand that I might be contacted to be interviewed about my experiences of being involved in this trial, which will be audio recorded but only used for research purposes.
9. This project is funded by a grant from the Victorian Cancer Agency (CPSRG19011).
10. I acknowledge that:
    a. The possible effects of participating in the research study have been explained to my satisfaction;
    b. I understand that my involvement in this study is entirely voluntary and will not affect my care in any way;
    c. I have been informed that I am free to withdraw from the study at any time without explanation or prejudice and to withdraw any unprocessed data I have provided;
    d. The study is for the purposes of research;

e. I have been informed that the confidentiality of the information I provide will be safeguarded subject to any legal requirements;

f. I have been informed that my consent form and information will be stored securely at the University of Melbourne and destroyed 5 years after publication of the study results;

g. I have been informed that a copy of the research findings will be forwarded to me, should I request it at the completion of the study;

h. In accordance with the law of Victoria, I understand that it is possible for data to be subject to subpoena, or freedom of information request.

**11.**	Contact from our Cancer in Primary Care Community Network<br>We have set up a network for people who would like to help shape our research. You may then be contacted in the future about joining the research network.<br>☐ Yes, I would like to be contacted in the future<br>☐ No, I would not like to be contacted in the future
**12.**	I would like to receive a summary of research results after the project has finished<br>☐ Yes<br>☐ No
**13.**	I consent to being audio-recorded<br>☐ Yes<br>☐ No

Name of participant: ______________________________________

Participant signature: _______________________________________ Date: ___________

ID:
Date:

Centre for Cancer Research,
Department of General Practice, University of Melbourne

BASELINE QUESTIONNAIRE

For entry into REDCap

Project:

The Bowel Cancer Prevention Study

Principal investigators:

Ms Shakira Milton, Prof Finlay Macrae, Prof Jon Emery

on behalf of the study investigators

Section 1: Your general demographic information

**1.**	What is your sex?	☐ Male	☐ Female	☐ Other
**2.**	What is your date of birth? (DD/MM/YYYY)	**___/___/ 19___**		
**3.**	Which language do you mainly speak at home?	☐ English<br>☐ Arabic<br>☐ Cantonese<br>☐ German<br>☐ Greek<br>☐ Italian<br>☐ Turkish<br>☐ Hindi<br>☐ Punjabi<br>☐ Macedonian<br>☐ Mandarin<br>☐ Spanish<br>☐ Vietnamese<br>☐ Other (please specify)<br><br>______________________		

**4.**	Which country were you born in?		
**5.**	How much formal education have you completed?	☐ Never completed high school<br>☐ Completed high school only<br>☐ TAFE qualification or similar<br>☐ University degree or higher	
**6.**	Do you live alone?	☐ Yes	☐ No
**7.**	If no, please state who you live with (e.g. wife, husband, partner, family members, friends)		
**8.**	Do you currently take any medications daily?	☐ Yes	☐ No

9.	If yes, how many tablets in total do you take daily? (excluding vitamins)	
10.	What is your phone number?	
11.	What is your email address?	
12.	How would you like to complete the follow up questionnaires?	☐ By post<br>☐ By email<br>☐ By phone
13.	If by post, what is your address?	

Section 2:

In this part of the survey we will be asking you some questions about your experience and feelings about numbers. Your answers to the following questions will help us a great deal in understanding how people use and understand numbers.

For each of the following questions, please check the box that best reflects how good you are at doing the following things:

1. How good are you at working with fractions?					
☐1	☐2	☐3	☐4	☐5	☐6
Not at all good					Extremely good
2. How good are you at working with percentages?					
☐1	☐2	☐3	☐4	☐5	☐6
Not at all good					Extremely good
3. How good are you at calculating a 15% tip?					
☐1	☐2	☐3	☐4	☐5	☐6
Not at all good					Extremely good
4. How good are you at figuring out how much a shirt will cost if it is 25% off?					
☐1	☐2	☐3	☐4	☐5	☐6
Not at all good					Extremely good

For each of the following questions, please check the box that best reflects your answer:

1. When reading the newspaper, how helpful do you find tables and graphs that are parts of a story?

❑1	❑2	❑3	❑4	❑5	❑6
Always prefer words					Always prefer numbers

2. When people tell you the chance of something happening, do you prefer that they use words ("it rarely happens") or numbers ("there's a 1% chance")?

❑1	❑2	❑3	❑4	❑5	❑6
Always prefer words					Always prefer numbers

3. When you hear a weather forecast, do you prefer predictions using percentages (e.g., “there will be a 20% chance of rain today”) or predictions using only words (e.g., “there is a small chance of rain today”)?

❑1	❑2	❑3	❑4	❑5	❑6
Always prefer percentages					Always prefer words

4. How often do you find numerical information to be useful?

❑1	❑2	❑3	❑4	❑5	❑6
Never					Very often

Section 3: Family History of Bowel Cancer:

**1.**	Have any of your close relatives (parent, brother sister, children) had bowel cancer before 55 years of age?	☐ Yes	☐ No	☐ Not sure
**2.**	Do you have more than one relative who had bowel cancer at any age? Please think about your parents, children, brothers, sisters, grandparents, aunts, uncles, nieces, nephews and grandchildren.	☐ Yes	☐ No	☐ Not sure

Section 4: Cardiovascular Risk:

**1.**	Have any of your close relatives had a heart attack, angina or stroke?	☐ Yes	☐ No	☐ Not sure
**2.**	Do you have diabetes?	☐ Yes	☐ No	☐ Not sure
**3.**	Do you take medication for high blood pressure?	☐ Yes	☐ No	☐ Not sure
**4.**	Have you ever been told by a doctor you have cholesterol?	☐ Yes	☐ No	☐ Not sure
**5.**	Do you currently or have you ever smoked cigarettes?	☐ Yes	☐ No	☐ Not sure

Thank you very much for completing this questionnaire

File D. Control video

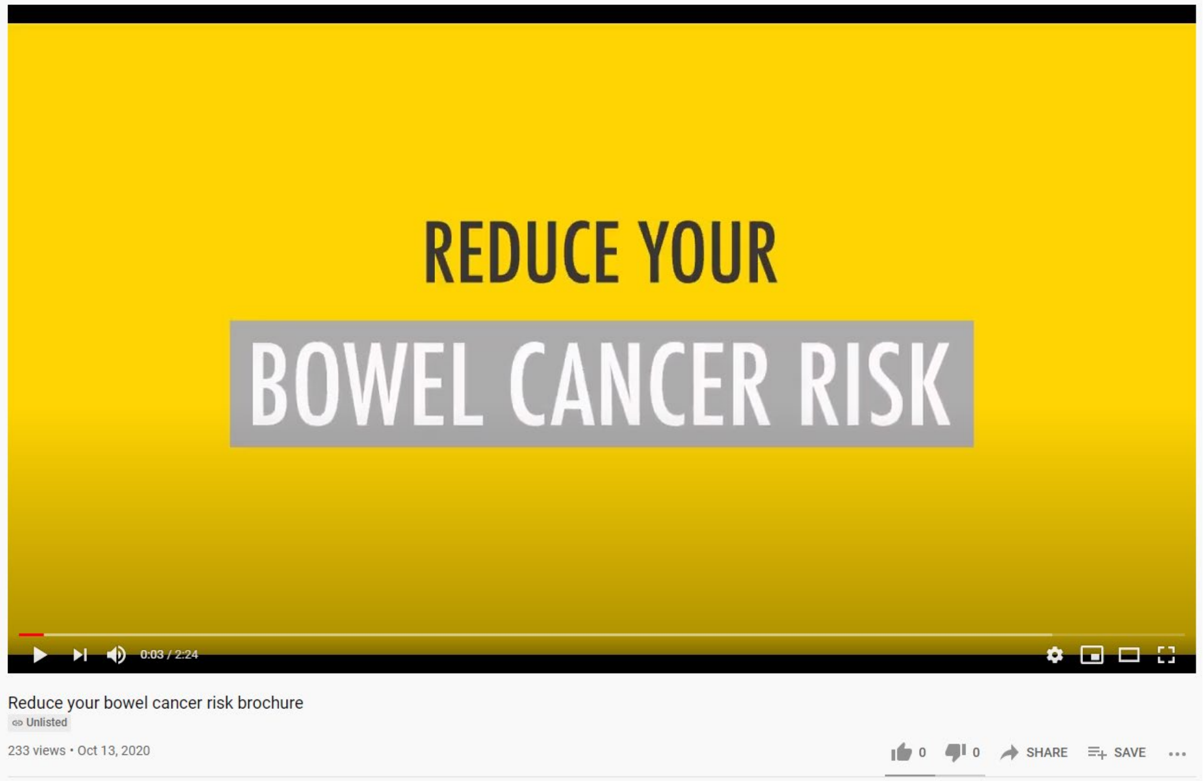

https://youtu.be/BzGHxV4-Yw0

File E. Control email brochure

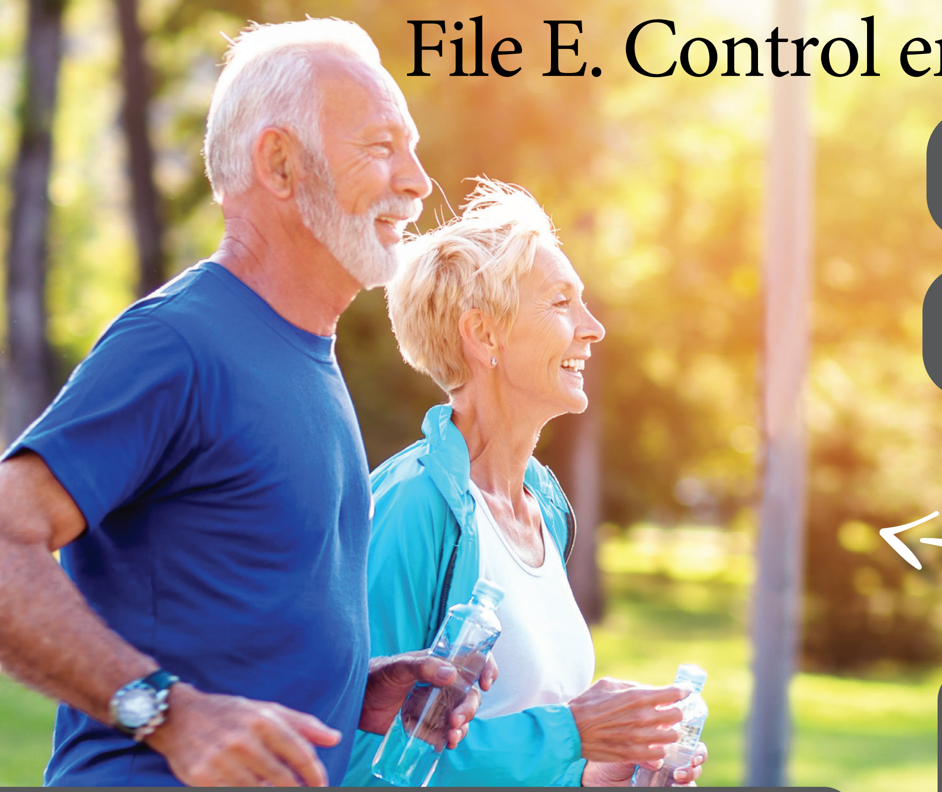

Reduce your bowel cancer risk

Maintain a healthy weight

Key tips!

- ✓ Reduce your waistline to under 94cm for men and 80cm for women
- ✗ Avoid food and drinks that are high in fats and sugars
- ✓ Limit alcoholic drinks
- ✓ Choose fish, poultry or vegetarian options instead of red meat for some meals

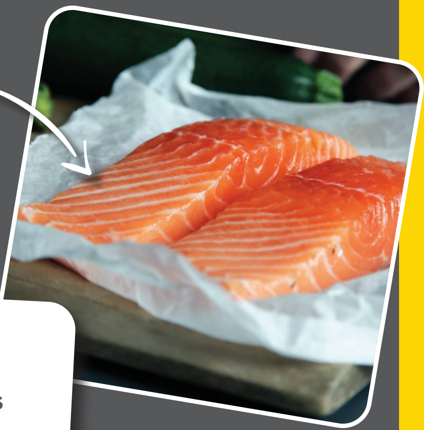

DID YOU KNOW

Nearly 2 in 3 Australian adults (63%) are overweight or obese

Speak to your GP about taking aspirin

Taking a low dose of aspirin every day for at least 2 ½ years decreases your risk of developing bowel cancer as well as dying from bowel cancer

Please note: the possible harms of aspirin use include a higher than normal risk of bleeding in the stomach or brain. Please discuss with your GP before taking aspirin.

Be physically active!

Key tips!

- ✓ Be physically active and elevate your heart rate every day in any way for 30 minutes or more.
- ✓ Walk or cycle instead of driving to the shops or work
- ✓ Use the stairs
- ✓ Meet a friend and play a sport together
- ✓ Take regular breaks at work

Check for unusual bowel habits & screen

Key tips!

- ✓ Complete the FOBT poo kit when it is delivered to you every two years from the age of 50 years

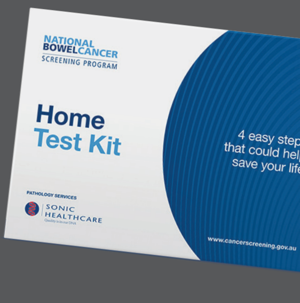

Eat a healthy diet, limit red & processed meat

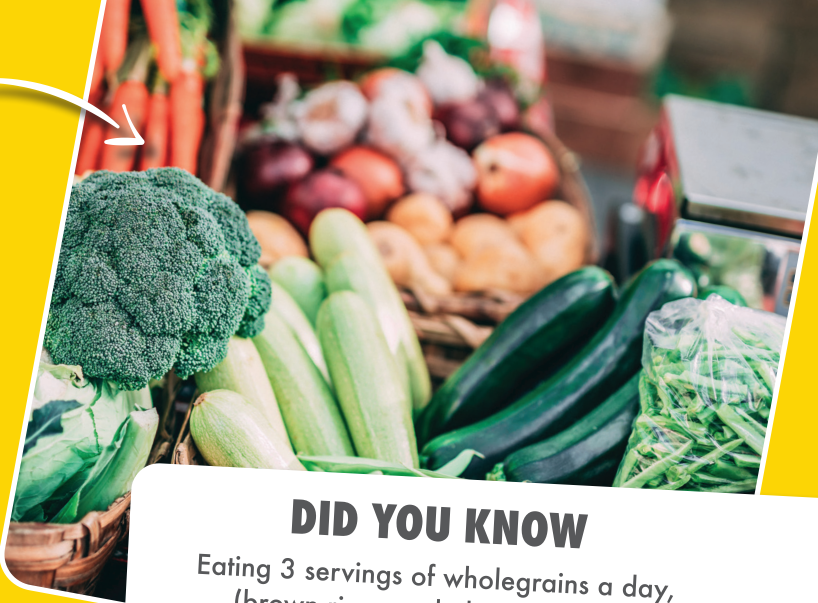

Key tips!

- ✓ Aim to eat 2 serves of fruit and 5 serves of vegetables daily
- ✗ Avoid eating processed meats such as bacon,ham, salami and sausages
- ✓ If you eat red meat, limit the amount to 500 grams cooked per week

DID YOU KNOW

Eating 3 servings of wholegrains a day, (brown rice or wholemeal bread) can reduce your risk of bowel cancer

DID YOU KNOW

Eating too much red meat has been linked with an increased risk of bowel cancer

Quit smoking

Key tips!

- ✓ Quit smoking
- ✗ Avoid exposure to tobacco smoke

DID YOU KNOW

Smoking 40 cigarettes (two packs) per day increases your risk of bowel cancer by around 40% and nearly doubles the risk of bowel cancer death

Call Quitline on
13 78 48
or visit
www.quit.org.au

Eat dairy products & take calcium supplements

Key tips!

- ✓ Include dairy products like low-fat milk, yoghurt and cheese in your diet
- ✓ Speak to your GP if you are lactose intolerant about what calcium supplement to take

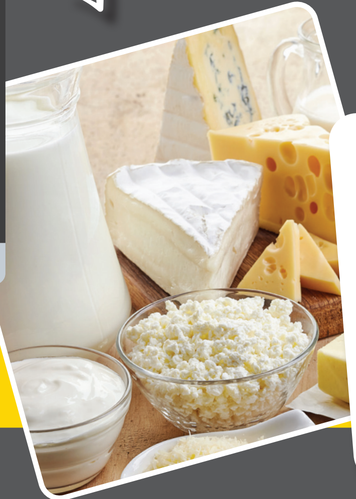

DID YOU KNOW

Having 400 grams of dairy products per day decreases your bowel cancer risk by 13% and having 200 grams of milk or 200mg of dietary calcium per day decreases risk by 6%

Drink less & live more

Key tips!

- ✓ Drink no more than two standard drinks per day

If you choose to drink alcoholic drinks, limit to special occasions

One standard drink equals

**ONE GLASS OF BEER 285 ml of beer**	**ONE SMALL GLASS OF WINE 100 ml of wine**	**ONE MEASURE OF SPIRITS 30 ml of spirits**

DID YOU KNOW

Almost 1 in 5 Australian adults (18%) consume more than 2 standard drinks per day

www.cutyourcancerrisk.org.au/how-to-cut-cancer-risk | www.bowelcanceraustralia.org/modifiable-risk-factors

File F. Teletrial decision aid for email

ARE YOU A MAN BETWEEN THE AGES OF 50 - 70?

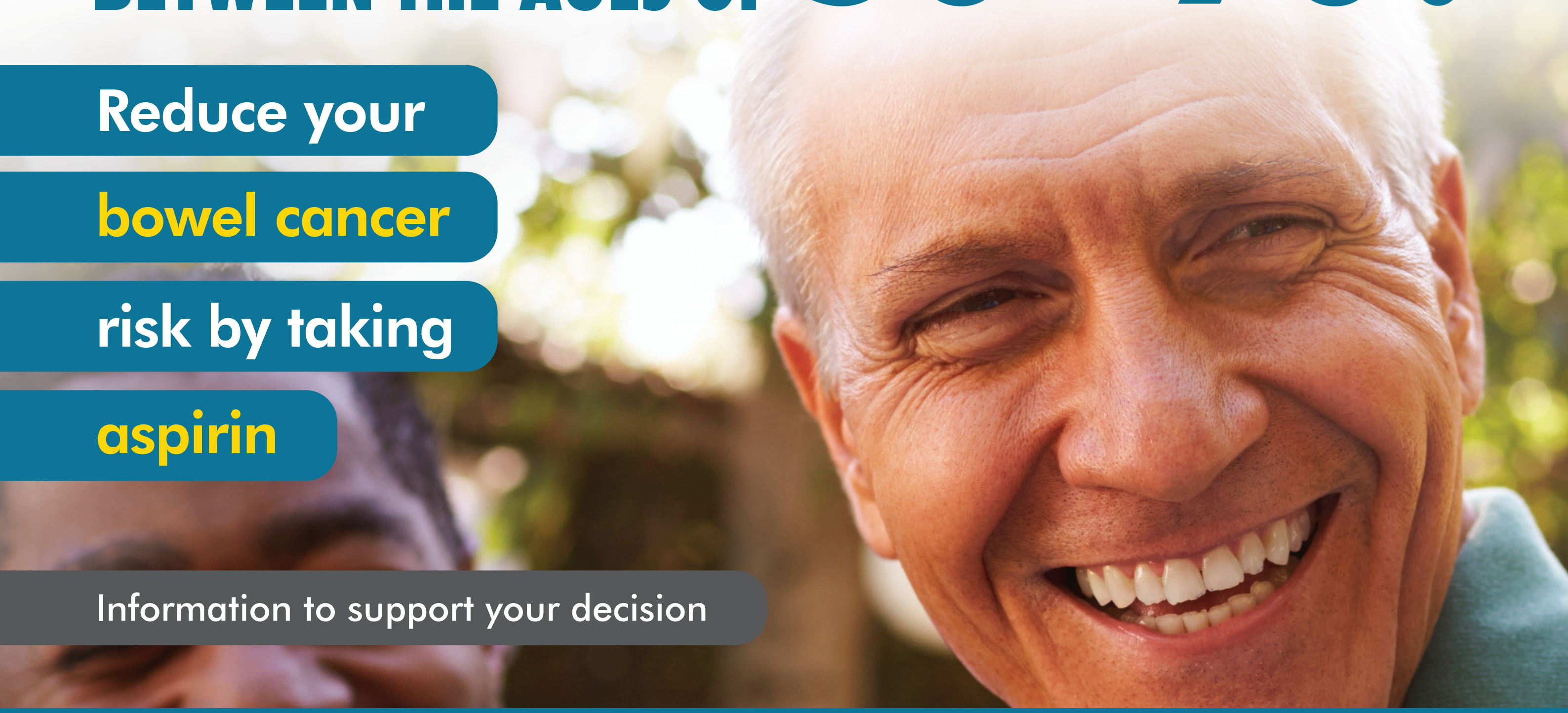

Reduce your **bowel cancer** risk by taking **aspirin**

Information to support your decision

Aspirin can reduce your risk of **bowel cancer** BY UP TO **25%**

Cancer Council Australia recommends that, if you are between the ages of 50 and 70, speak to your GP about taking low dose aspirin (100-300 mg every day) to prevent bowel cancer.

Cancer Council Australia

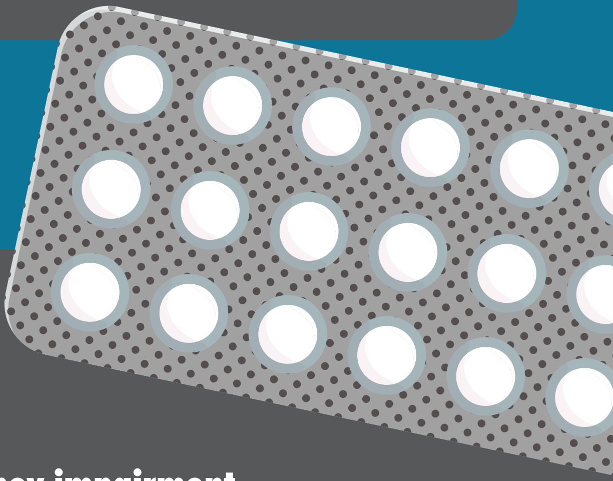

Aspirin may not be appropriate if you have...

- ✖ Indigestion
- ✖ Untreated high blood pressure
- ✖ Stomach ulcers
- ✖ Blood thinning medication e.g. Warfarin
- ✖ Kidney impairment
- ✖ Untreated H. pylori
- ✖ A bleeding disorder

Speak to your GP today

This brochure will help you

- Understand the benefits and side-effects of taking low dose aspirin.
- Decide whether taking aspirin is the right decision for you.

How much aspirin should I take?

- 100 – 300 mg (e.g. one baby aspirin per day).
- For a minimum of 2½ to 5 years.

Remember to:

- Talk to your GP before taking aspirin.
- Continue regular screening with the poo test (aka FOBT kit) that you receive in the post from the National Bowel Cancer Screening Program.

Aspirin can reduce your risk of bowel cancer, heart disease and stroke

but can increase your risk of bleeding from the stomach and gut.

This figure shows what would happen to **10,000 Australian men between the ages of 50 to 70** after 10 years if they did or did not take aspirin.

INCIDENCES OF THE FOLLOWING

	BOWEL CANCER	HEART ATTACK	STROKE	DEATH FROM ALL CAUSES	BLEEDING FROM GUT & STOMACH
NO ASPIRIN	178	743	596	1075	104
ASPIRIN	134	707	591	1027	129
DIFFERENCE	44 FEWER	36 FEWER	5 FEWER	48 FEWER	25 MORE

File G. Video decision aid for males

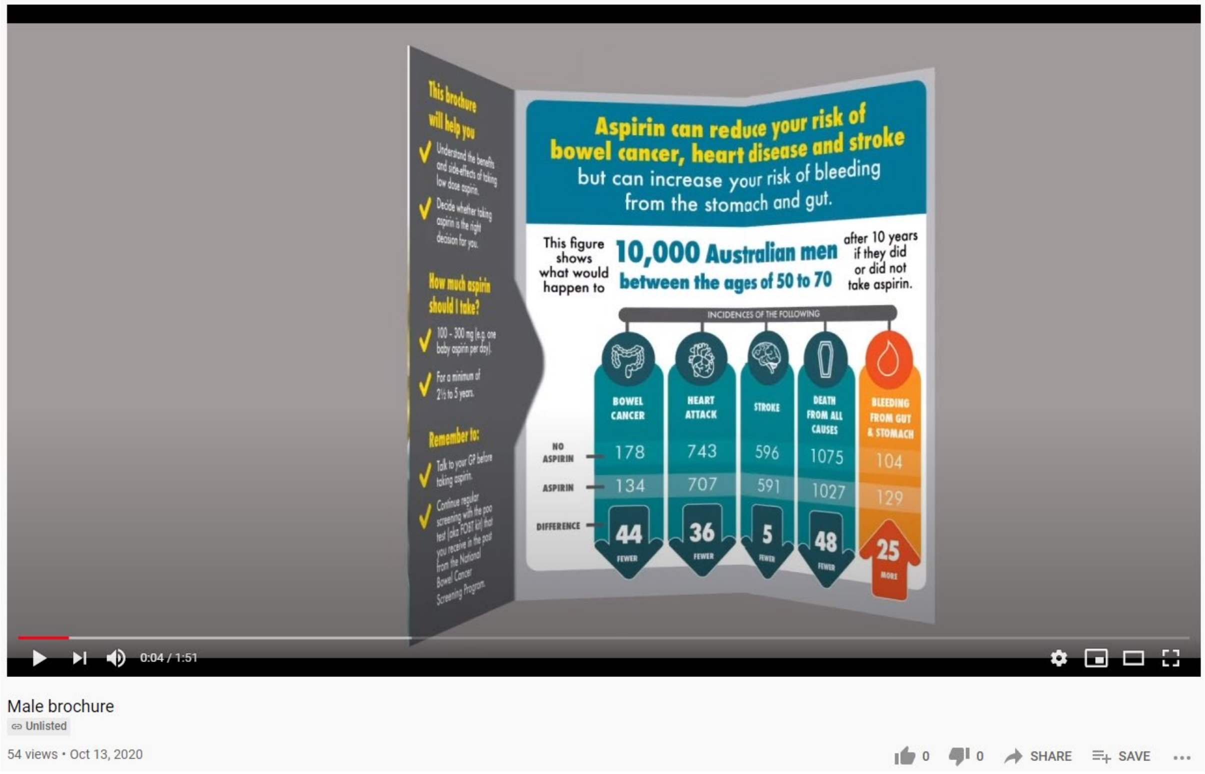

https://youtu.be/p_Ey908EApE

File H. Video decision aid for females

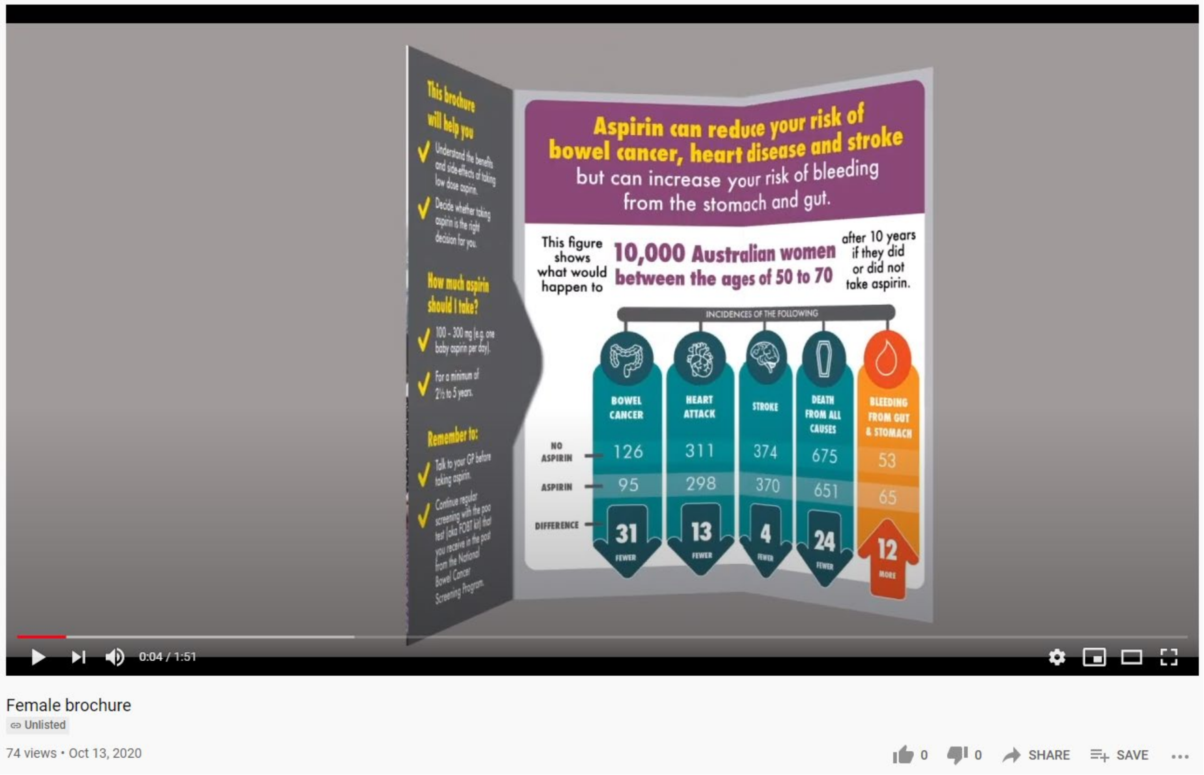

https://youtu.be/cDf_3mlJRoU

ID:
Date:

The Bowel Cancer Prevention Study

QUESTIONNAIRE

One month

Please answer all the questions.

Your answers will be treated as confidential and will only be used for research purposes.

All areas that need to be answered are shaded in grey as in the example to the right.

0.0 This is an example question

- [x] Yes
- [ ] No

If you prefer to complete the questionnaire online, please email:

sita-trial@unimelb.edu.au

Centre for Cancer Research,
Department of General Practice

Principal investigators: Prof Jon Emery, Ms Shakira Milton, Prof Finlay Macrae on behalf of the study investigators

THE UNIVERSITY OF MELBOURNE

SECTION 1: In this section, we ask about things you have done since you joined the study to reduce your chances of getting bowel cancer. Since I joined this study, I have:

PLEASE TICK YOUR RESPONSE		YES	NO
1	Changed my diet		
2	Talked to my GP about quitting smoking		
3	Quit smoking		
4	Talked to my GP about completing a bowel cancer screening test (the FOBT or poo test)		
5	Completed a bowel cancer screening test (the FOBT or poo test)		
6	Talked to my GP about having a colonoscopy		
7	Had a colonoscopy		
8	Talked to my GP about taking aspirin		

PLEASE TICK YOUR RESPONSE

9 Taking aspirin (5 or more out of 7 days in a week)

- ☐ Yes, I am currently taking aspirin
- ☐ I started then stopped taking aspirin
- ☐ No, I haven't taken aspirin in the last month (If no, skip to section 2)

10 If you answered 'yes' or 'started then stopped taking aspirin' to the last question, what dose of aspirin do you/ did you take each day?

- ☐ 100 mg
- ☐ 300 mg
- ☐ Other (please specify)

11 What are your reasons for taking aspirin? **(TICK AS MANY AS APPLY)**

- ☐ To reduce my risk of a heart attack
- ☐ To reduce my risk of a stroke
- ☐ To reduce my risk of bowel cancer
- ☐ Other (please specify)

____

12 Have you experienced side-effects while taking aspirin?

☐ Yes ☐ No

13 Which side-effects have you experienced? **(TICK AS MANY AS APPLY)**

- ☐ Nausea
- ☐ Easy bruising
- ☐ Indigestion
- ☐ Bleeding
- ☐ Other (please specify)

____

SECTION 2:

PLEASE TICK YOUR RESPONSE

14 Of the following choices to reduce my chances of bowel cancer, I prefer to (select one);

- ☐ Change my diet
- ☐ Do the bowel cancer screening test (the FOBT or poo test)
- ☐ Take aspirin
- ☐ Unsure

PLEASE TICK YOUR RESPONSE

Considering the option you prefer in the previous question, please answer the following questions:

		STRONGLY DISAGREE	DISAGREE	NEITHER AGREE OR DISAGREEE	AGREE	STRONGLY AGREE
15	I know which options are available to me					
16	I know the benefits of each option					
17	I know the risks and side effects of each option					
18	I am clear about which benefits matter most to me					
19	I am clear about which risks and side effects matter most					
20	I am clear about which is more important to me (the benefits or the risks and side effects)					
21	I have enough support from others to make a choice					
22	I am choosing without pressure from others					
23	I have enough advice to make a choice					
24	I am clear about the best choice for me					
25	I feel sure about what to choose					
26	This decision is easy for me to make					
27	I feel I have made an informed choice					
28	My decision shows what is important to me					
29	I expect to stick with my decision					
30	I am satisfied with my decision					

SECTION 3: Now we would like to ask you some questions about taking aspirin to reduce your chances of getting various conditions. Even if you don't take aspirin, we'd like to get your opinion on these issues. For the following statements, please state whether they are true or false.

PLEASE TICK YOUR RESPONSE

For the following statements, please state whether they are true or false.
(You don't need to look these up, give your best answer.)

		TRUE	FALSE	UNSURE
31	Taking aspirin daily can increase my risk of bleeding			
32	Taking aspirin daily can increase my risk of dementia			
33	Taking aspirin daily can reduce my risk of heart attacks and strokes			
34	Taking aspirin daily can reduce my risk of bowel cancer			
35	People who have had angina or a heart attack should consider taking aspirin			
36	People who have had a stomach ulcer should consider taking aspirin			
37	People who have several close relatives with bowel cancer should consider taking aspirin			
38	Healthy people aged 50-70 years should consider taking aspirin			
39	Aspirin reduces my chance of bowel cancer if I take it daily for at least a year			
40	Aspirin reduces my chance of bowel cancer if I take it daily for at least 2½ years			
41	Aspirin doesn't have any effect on my chance of getting bowel cancer			

42 What are the common side effects of aspirin? Please list as many as you can:

SECTION 4: For the following questions, please circle the number from 1 to 7 on the scale that best describes how you feel at the moment.

For example, in question (a) if you thought taking aspirin would be very beneficial, you would circle 1. If you thought it would be slightly beneficial, you would circle 3 and if you thought it would be slightly harmful you would circle 5.

Please read the scale for each question.

PLEASE CIRCLE YOUR RESPONSE

For me, taking aspirin to reduce my bowel cancer risk is:

		VERY	QUITE	SLIGHTLY	NEITHER	SLIGHTLY	QUITE	VERY	
a)	Beneficial	1	2	3	4	5	6	7	Harmful
b)	Important	1	2	3	4	5	6	7	Unimportant
c)	Good thing	1	2	3	4	5	6	7	Bad thing
d)	Pleasant	1	2	3	4	5	6	7	Unpleasant

Thank you

FOR COMPLETING THIS QUESTIONNAIRE

WE WILL SEND YOU THE LAST QUESTIONNAIRE IN FIVE MONTHS

The Bowel Cancer Prevention Study

ID:
Date:

QUESTIONNAIRE

Six months

Please answer all the questions.

Your answers will be treated as confidential and will only be used for research purposes.

All areas that need to be answered are shaded in grey as in the example to the right.

0.0 This is an example question

- [x] Yes
- [ ] No

If you prefer to complete the questionnaire online, please email:

sita-trial@unimelb.edu.au

Centre for Cancer Research,
Department of General Practice

Principal investigators: Prof Jon Emery, Ms Shakira Milton, Prof Finlay Macrae on behalf of the study investigators

THE UNIVERSITY OF MELBOURNE

SECTION 1: In this section, we ask about things you have done since you joined the study to reduce your chances of getting bowel cancer. Since I joined this study, I have:

	PLEASE TICK YOUR RESPONSE	YES	NO
1	Changed my diet		
2	Talked to my GP about quitting smoking		
3	Quit smoking		
4	Talked to my GP about completing a bowel cancer screening test (the FOBT or poo test)		
5	Completed a bowel cancer screening test (the FOBT or poo test)		
6	Talked to my GP about having a colonoscopy		
7	Had a colonoscopy		
8	Talked to my GP about taking aspirin		

PLEASE TICK YOUR RESPONSE

9 Taking aspirin (5 or more out of 7 days in a week)

- ☐ Yes, I am currently taking aspirin
- ☐ I started then stopped taking aspirin
- ☐ No, I haven't taken aspirin in the last month (If no, skip to question 14)

10 If you answered 'yes' or 'started then stopped taking aspirin' to the last question, what dose of aspirin do you/ did you take each day?

- ☐ 100 mg
- ☐ 300 mg
- ☐ Other (please specify)

11 What are your reasons for taking aspirin? **(TICK AS MANY AS APPLY)**

- ☐ To reduce my risk of a heart attack
- ☐ To reduce my risk of a stroke
- ☐ To reduce my risk of bowel cancer
- ☐ Other (please specify)

____

PLEASE TICK YOUR RESPONSE

12 Have you experienced side-effects while taking aspirin?

- ☐ Yes
- ☐ No

13 Which side-effects have you experienced?
(TICK AS MANY AS APPLY)

- ☐ Nausea
- ☐ Easy bruising
- ☐ Indigestion
- ☐ Bleeding
- ☐ Other (please specify) ______________________

14 I stopped taking aspirin OR I don't take aspirin because:
(TICK AS MANY AS APPLY)

- ☐ It's something I've not considered
- ☐ My doctor advised against it
- ☐ I am at increased risk of bleeding
- ☐ I experienced a side-effect
- ☐ Other (please specify) ______________________

Thank you

FOR COMPLETING THIS QUESTIONNAIRE
